# Gastric Metastasis of Uterine Leiomyosarcoma Detected During Surveillance Endoscopy and Resected by Endoscopic Submucosal Dissection After Multiple Metachronous Metastases: A Case Report

**DOI:** 10.1002/deo2.70370

**Published:** 2026-07-07

**Authors:** Yuiko Nagasawa, Takahiro Hiratsuka, Daisuke Minezaki, Wataru Miyoshino, Ryo Ogawa, Tomonori Akagi, Hidefumi Shiroshita, Yoshitake Ueda, Kazuhiro Mizukami, Masafumi Inomata

**Affiliations:** ^1^ Department of Gastroenterological and Pediatric Surgery Faculty of Medicine Oita University Japan; ^2^ Center For Community Medicine Faculty of Medicine Oita University Japan; ^3^ Department of Gastroenterology Faculty of Medicine Oita University Japan; ^4^ Department of Advanced Medical Personnel Nurturing Faculty of Medicine Oita University Japan

**Keywords:** endoscopic submucosal dissection, gallbladder metastasis, gastric metastasis, sigmoid colon metastasis, uterine leiomyosarcoma

## Abstract

Uterine leiomyosarcoma (uLMS) is a rare, aggressive uterine malignancy that usually metastasizes hematogenously, whereas gastrointestinal involvement is uncommon. A 69‐year‐old woman with uLMS treated by hysterectomy and adjuvant chemotherapy developed metachronous lung, sigmoid colon, gallbladder, and abdominal wall metastases. After treatment of these metastases, periodical surveillance endoscopy revealed a 15‐mm subepithelial lesion with a shallow central depression in the lower gastric body. Endoscopic ultrasonography showed a well‐defined submucosal lesion without muscularis propria invasion, and biopsy confirmed metastatic leiomyosarcoma. Endoscopic submucosal dissection (ESD) was performed, achieving en bloc resection without adverse events. This rare metachronous combination of gastric, colonic, and gallbladder metastases highlights that incidental gastric metastasis may occur during prolonged disease courses and suggests that ESD may be a useful minimally invasive option for local control of small gastric metastases.

## 1 Introduction

Uterine leiomyosarcoma (uLMS) is a high‐grade malignant mesenchymal tumor accounting for 1%–2% of uterine malignancies, with an annual incidence of approximately 0.3–0.4 per 100,000 women [1, 2]. It is characterized by aggressive behavior, with reported overall recurrence rates of 45%–70% during the disease course and poor prognosis once metastatic disease develops [1, 2, 3]. In a retrospective cohort of 113 patients, Tirumani et al. reported that distant metastases occurred in 81.4% of the cases, predominantly via hematogenous spread to the lungs (74%), followed by the peritoneum (41%), bone (33%), and liver (27%) [4].

Although gastric, colonic, and gallbladder metastases from uLMS have each been described individually, their metachronous combination in a single patient appears to be extremely uncommon. This report aimed to describe this rare presentation of gastric metastasis mimicking a subepithelial lesion and to highlight the clinical role of endoscopic submucosal dissection (ESD) as a minimally invasive approach for diagnosis and local management in patients with prolonged metastatic courses.

## 2 Case Report

**FIGURE 1:**
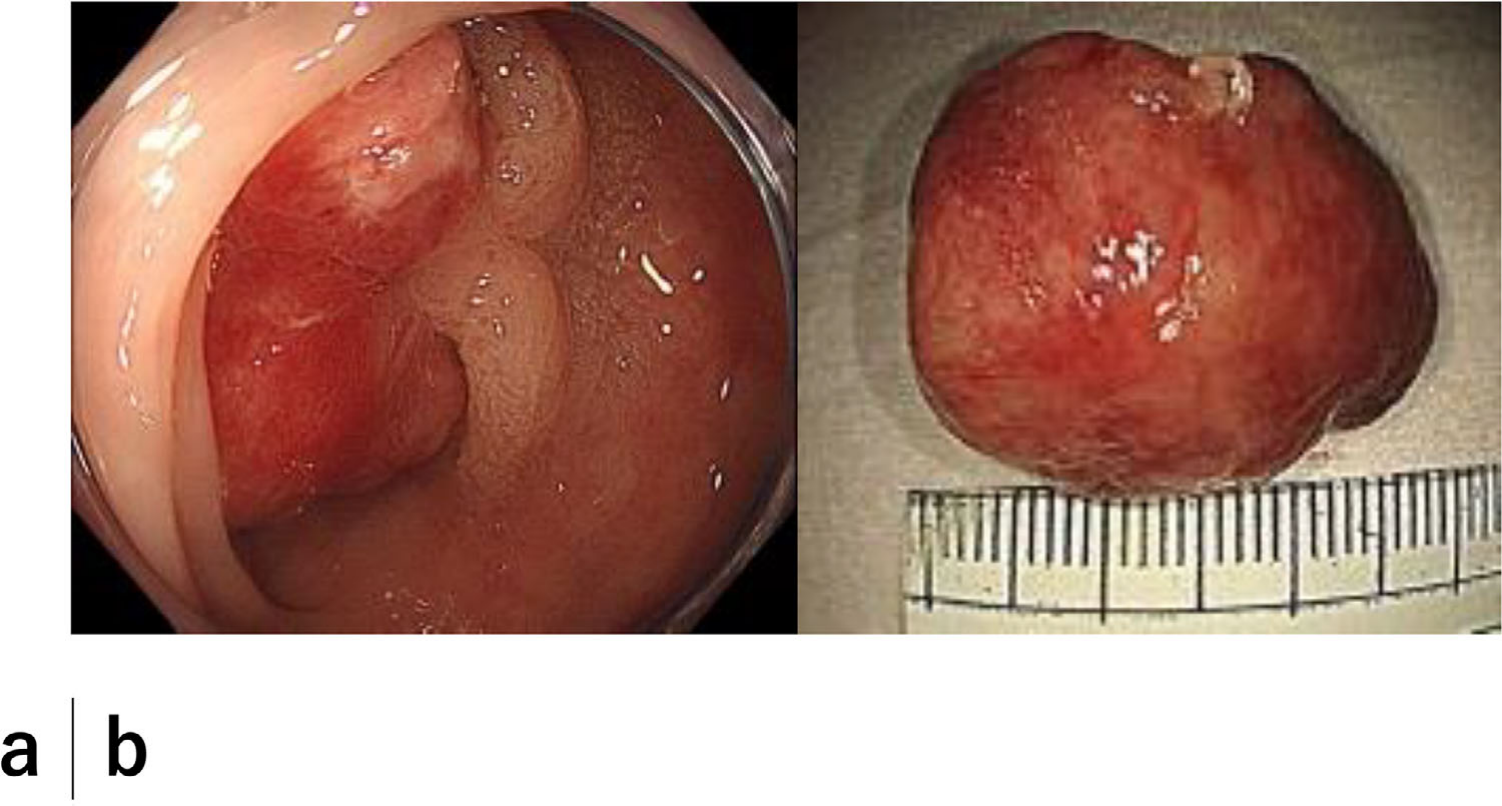
Sigmoid colonic metastasis. (a) Colonoscopic view of a polypoid lesion in the sigmoid colon. (b) Resected specimen obtained by endoscopic mucosal resection.

**FIGURE 2:**
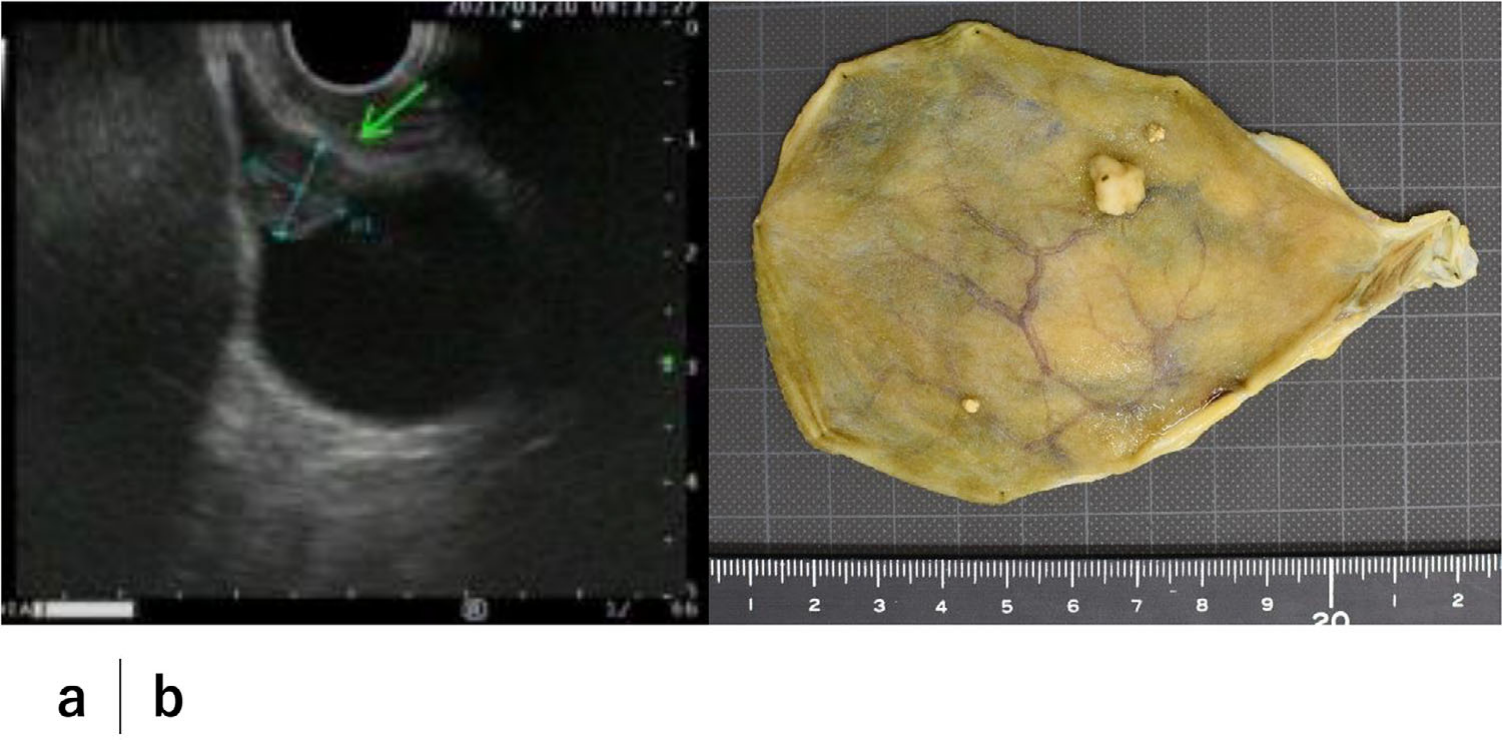
Gallbladder metastasis. (a) Endoscopic ultrasonography showing a solid intraluminal lesion in the gallbladder. (b) Gross specimen obtained after laparoscopic cholecystectomy.

**FIGURE 3:**
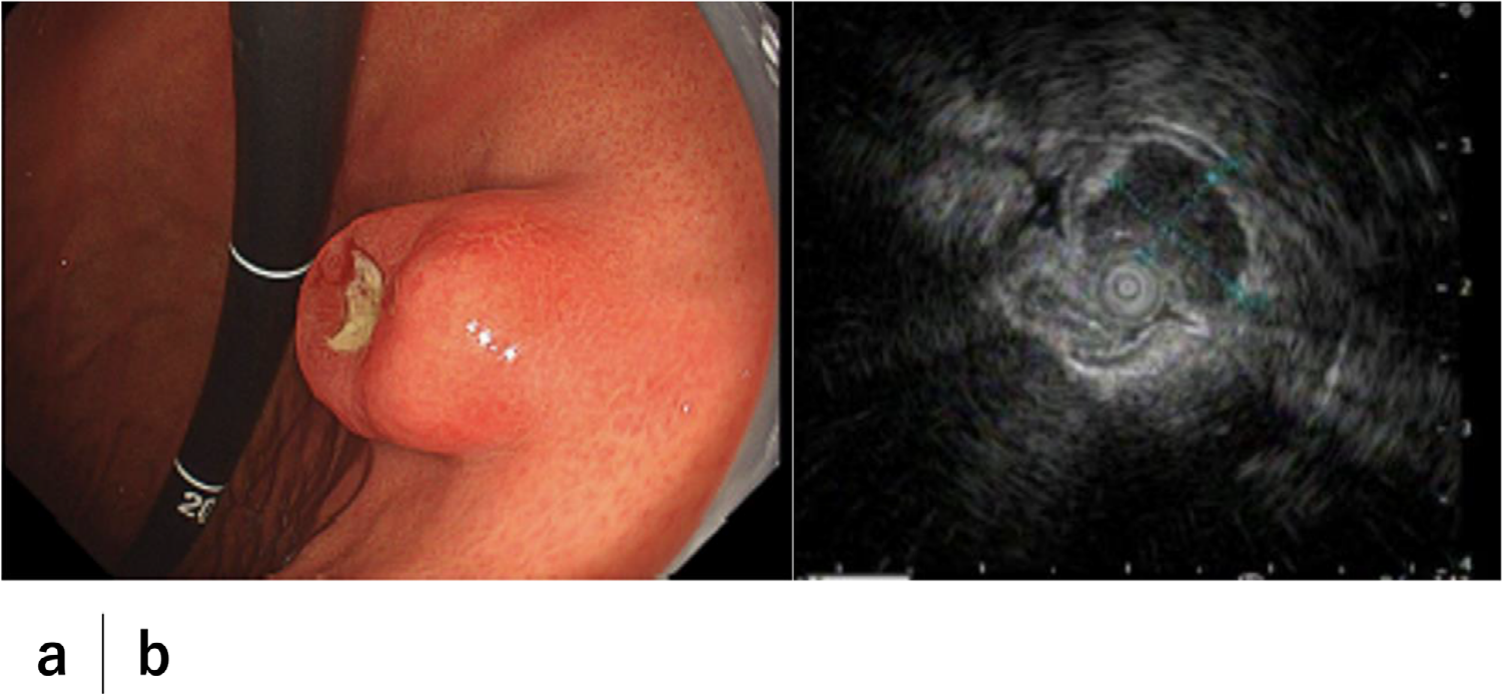
Gastric metastasis of uterine leiomyosarcoma. (a) White‐light endoscopy showing a 15‐mm subepithelial lesion with a shallow central depression on the posterior wall of the lower gastric body. (b) Endoscopic ultrasonography showing a well‐defined lesion located mainly in the third layer with preservation of the fourth layer.

A 69‐year‐old woman had undergone total hysterectomy and bilateral salpingo‐oophorectomy for uLMS 15 years earlier, followed by pelvic and para‐aortic lymphadenectomy and adjuvant chemotherapy.  During long‐term follow‐up, she developed metachronous metastases in the lungs, sigmoid colon, gallbladder, and abdominal wall. Each lesion was detected either incidentally or during evaluation of disease progression and was managed with a combination of local treatment and systemic chemotherapy according to disease extent and clinical context (Table 1). The sigmoid colonic metastasis was treated by endoscopic mucosal resection (Figure 1), and the gallbladder metastasis by laparoscopic cholecystectomy (Figure 2). After treatment of these metachronous metastases, esophagogastroduodenoscopy performed as part of periodical surveillance revealed a 15‐mm subepithelial lesion with a shallow central depression on the posterior wall of the lower gastric body (Figure 3a).

**TABLE 1:** Clinical course and treatments of metachronous metastases.

Time from initial surgery	Metastatic site	Detection trigger	Maximum diameter (mm)	Differential diagnosis	Endoscopic findings	Local treatment	Systemic therapy (no. of cycles)
One month	—	—	—	—	—	—	DG (6)
Nine years	Lung	Surveillance CT/cough	20	Metastatic tumor Lung cancer	—	Thoracoscopic resection	Dox (6)
Eleven years	Sigmoid colon	Hematochezia	30	Juvenile polyps Hamartomatous polyps Metastatic tumor	CS: Pedunculated, spherical polypoid lesion. Smooth surface with marked erythema. EUS: the mucosal and superficial submucosal layers, with no evidence of deep submucosal invasion	EMR	—
Eleven years	Gallbladder	Surveillance CT	8	Gallbladder cancer	EUS: Type Isp‐like elevated lesion, hypoechoic, broad‐based; tumor invasion limited to the superficial subserosal layer. CH‐EUS: Early arterial‐phase hyperenhancement indicating rich tumor vascularity.	Laparoscopic cholecystectomy	DG (6)
Twelve years	Abdominal wall	Palpable mass	20	Lipoma, dermatofibroma Sebaceous cyst Cutaneous lymphoma	—	Surgical excision	DG(6)
15 years	Stomach	Screening endoscopy	16	Metastatic tumor GIST	EGD: subepithelial lesion with a shallow central depression on the posterior wall of the lower gastric body. EUS: well‐defined, hyperechoic mass arising from the submucosal.	ESD	Planning

**Abbreviations**: CS: colonoscopy; CT, computed tomography; DG, Docetaxel + Gemcitabine; Dox, Doxorubicin; EMR, endoscopic mucosal resection; ESD, endoscopic submucosal dissection; EUS, endoscopic ultrasonography; uLMS, uterine leiomyosarcoma.

Endoscopic ultrasonography demonstrated a well‐defined lesion located mainly in the submucosal layer, with preservation of the muscularis propria and no evidence of deep invasion (Figure 3b).

**FIGURE 4:**
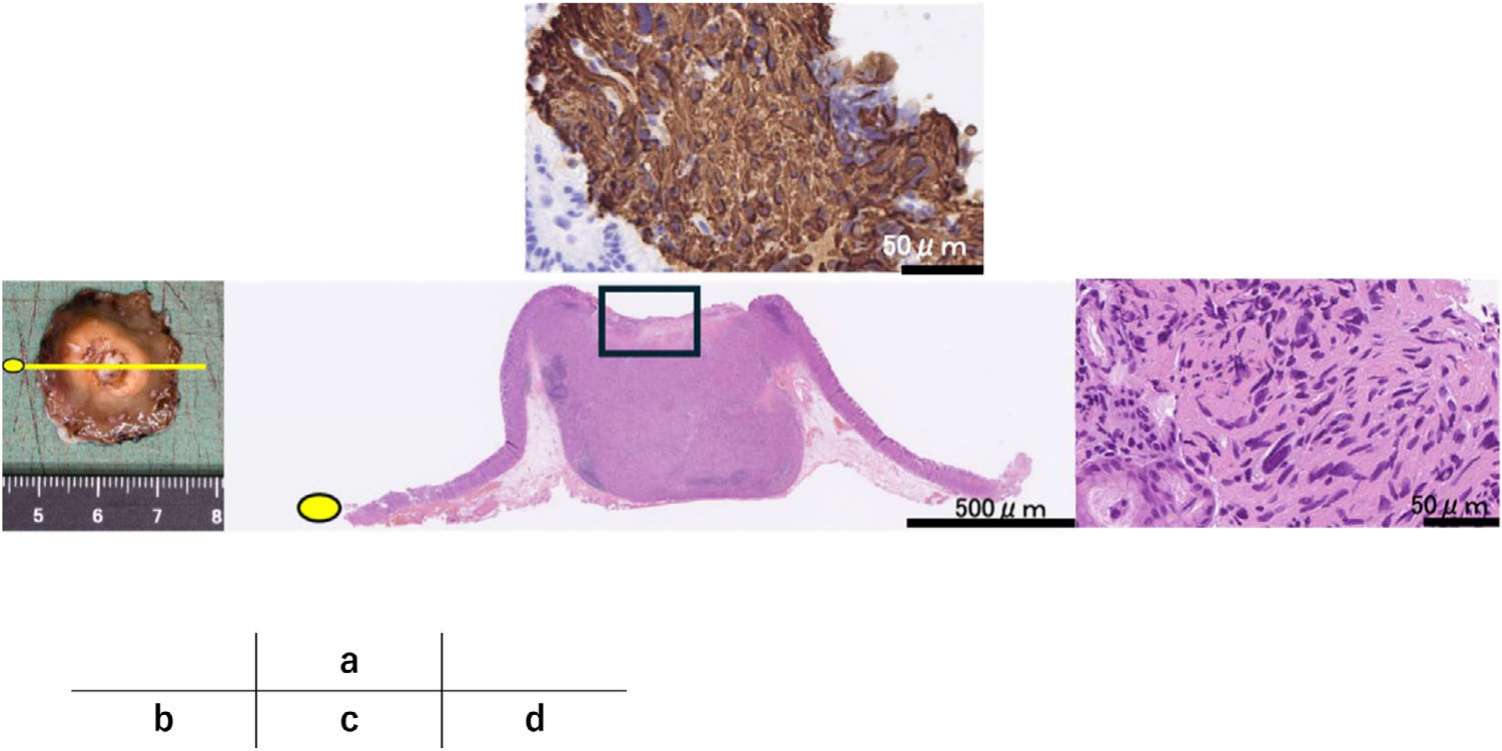
Histopathological findings of the gastric metastatic lesion. (a) Desmin immunostaining of the pretreatment biopsy specimen (×20) showing diffuse positivity in atypical spindle cells. (b) Gross appearance of the endoscopic submucosal dissection (ESD) specimen. (c) Loupe view of the ESD specimen showing a well‐demarcated subepithelial lesion (H&E staining). (d) High‐power view of the ESD specimen (H&E staining, ×20) showing atypical spindle cells arranged in fascicles.

Biopsy specimens obtained from the depressed area showed proliferation of atypical spindle cells. Immunohistochemical staining of the biopsy specimen demonstrated positivity for desmin and alpha‐smooth muscle actin and negativity for S‐100 and CD34, supporting the diagnosis of metastatic leiomyosarcoma (Figure 4a and Figure S1). Given the patient's oncologic history of repeated metachronous metastases from uLMS, gastric metastasis was considered more likely than a primary gastric mesenchymal tumor.

At the time of detection of the gastric lesion, the patient had a performance status of 0, and no evidence of metastases to organs other than the stomach was identified. She was not receiving systemic chemotherapy at that time. Her most recent systemic chemotherapy had been six courses of docetaxel and gemcitabine for abdominal wall metastasis, and the gastric lesion was detected approximately 18 months after completion of this treatment.

Regarding treatment strategy, the patient's overall prognosis was considered to depend primarily on systemic disease control rather than on local treatment of the gastric lesion alone. However, the lesion was small, appeared to be confined mainly to the submucosal layer on endoscopic ultrasonography, and had already been strongly suspected to represent metastatic leiomyosarcoma on biopsy. In addition, en bloc resection enabled pathological assessment, reduced potential bleeding risk, and avoided delay in resuming systemic chemotherapy. Therefore, ESD was selected as a minimally invasive local treatment.

The lesion was marked endoscopically, and ESD was performed using a multibending endoscope. Submucosal dissection was performed using a retroflexed approach. Because no distinct capsule was identified, dissection was carefully advanced just above the muscularis propria. After en bloc resection, the mucosal defect was completely closed with endoscopic clips. The procedure was completed without adverse events, and the total procedure time was 31 min. The resected specimen measured 30 × 28 mm, and the tumor measured 16 × 12 mm.

Histopathological examination of the ESD specimen revealed a well‐demarcated subepithelial spindle cell lesion composed of atypical tumor cells with marked nuclear atypia arranged in fascicles (Figure 4b–d). Both horizontal and vertical margins were negative. These morphological findings were consistent with the diagnosis of metastatic uLMS that had been suggested by the biopsy‐based immunohistochemical findings.

At 2 months after ESD, no local recurrence was observed. Over the course of her disease, the clinical trajectory evolved from localized recurrence to a chronic systemic condition requiring sequential lines of chemotherapy, while local surgical or endoscopic interventions were selectively applied for organ‐limited metastases and local disease control.

## 3 Discussion

To our knowledge, reports describing metachronous gastric, colonic, and gallbladder metastases from uLMS in a single patient are extremely limited. Metastatic uLMS carries a poor prognosis. In a retrospective cohort of 113 patients, Tirumani et al. reported a median overall survival of 45 months, with 65% of patients deceased at analysis [4]. This case also showed an unusually long interval between the initial uLMS diagnosis and gastric metastasis, as distant metastases reportedly develop after a median of 7 months [4]. Compared with this typical pattern, gastric metastasis detected 15 years after the initial diagnosis appears to be an unusual delayed manifestation, likely reflecting the patient's prolonged disease course under sequential systemic and local treatments.

The long‐term clinical course in the present case cannot be explained by local treatment alone. At initial surgery, the primary tumor was confined to the uterus and classified as FIGO stage IB, without adnexal involvement, nodal metastasis, peritoneal dissemination, distant metastasis, or definite lymphovascular invasion. However, the tumor was large (10 cm), showed high mitotic activity (25/10 high‐power fields), and corresponded to FNCLCC grade 3, indicating aggressive pathological features despite localized disease.

Gastrointestinal metastases from uLMS are uncommon and are mainly reported as isolated cases. Documented sites include the stomach, small intestine, and colon, often presenting with bleeding or obstruction in advanced disease [5, 6]. The endoscopic morphology of gastric metastases from leiomyosarcoma appears to be variable. Previous reports have described submucosal tumor‐like lesions with erosion or ulceration, protruding lesions, shallow depressions, and, in some cases, multiple gastric lesions [5, 7]. In contrast, the present lesion was a solitary, small subepithelial lesion with a shallow central depression, detected in an asymptomatic patient during surveillance. These findings may mimic primary gastric subepithelial tumors, including gastrointestinal stromal tumor, leiomyoma, schwannoma, and primary gastric leiomyosarcoma; therefore, biopsy and immunohistochemical evaluation are essential for diagnosis. In previously reported cases, gastric metastases were often detected in the context of advanced systemic disease or symptomatic presentations such as bleeding or anemia, suggesting that gastric involvement generally reflects disseminated disease rather than an isolated curable lesion.

Although most reported gastric metastases from leiomyosarcoma have been treated surgically or palliatively because of bleeding, anemia, or advanced systemic disease [5, 7], the present lesion was small, solitary, and mainly confined to the submucosal layer on endoscopic ultrasonography. In this case, the 15‐mm subepithelial lesion was asymptomatic but showed central depression, raising concern for possible future bleeding. Because ongoing systemic therapy was expected to be required during the disease course, a minimally invasive strategy was essential. These features made en bloc endoscopic resection technically feasible and clinically reasonable for diagnostic confirmation, local control, and prevention of local complications. Although not curative, ESD provided both diagnostic confirmation and local control without surgical morbidity in the setting of prolonged metastatic disease.

The role of metastasectomy in uLMS remains controversial. Although evidence is not specific to uLMS alone, a systematic review of leiomyosarcoma, including uLMS, suggested that selected patients with limited and resectable metastases may benefit from complete resection [8]. However, metastatic uLMS often follows a recurrent and heterogeneous clinical course, requiring individualized multimodal management that integrates systemic therapy with local treatment when clinically appropriate [9]. Current guidelines emphasize long‐term follow‐up with individualized surveillance strategies [10]. In selected patients with repeated recurrences, endoscopic evaluation may therefore be clinically meaningful when indicated by symptoms, imaging findings, or the need for diagnostic confirmation.

This report has several limitations. Primary tumor specimens were unavailable for direct pathological comparison. In addition, long‐term follow‐up after ESD was limited, making it difficult to fully evaluate the long‐term clinical value of ESD in this setting. Nevertheless, ESD provided diagnostic confirmation and local control with minimal invasiveness in this patient with prolonged metastatic disease.

## Author Contributions

Conceptualization: **Yuiko Nagasawa**. Investigation: Yuiko Nagasawa, **Takahiro Hiratsuka**, **Daisuke Minezaki**, and **Wataru Miyoshino**. Supervision: **Tomonori Akagi**, **Hidefumi Shiroshita**, **Yoshitake Ueda**, **Ryo Ogawa**, **Kazuhiro Mizukami**, and **Masafumi Inomata**. Writing – original draft: **Yuiko Nagasawa**. Writing – review & editing: **Yuiko Nagasawa**, **Takahiro Hiratsuka**, **Daisuke Minezaki**, **Wataru Miyoshino**, **Tomonori Akagi**, **Hidefumi Shiroshita**, **Yoshitake Ueda**, **Ryo Ogawa**, **Kazuhiro Mizukami**, and **Masafumi Inomata**.

## Funding

This study received no specific grants from funding agencies in the public, commercial, or not‐for‐profit sectors. The authors have nothing to report.

## Ethics Statement

Ethics Committee approval was not required for this case report. All the procedures were conducted in accordance with the principles of the Declaration of Helsinki.

## Consent

Written informed consent for publication was obtained from the patient.

## Conflicts of Interest

The authors declare no conflicts of interest.

## Supporting information




**Figure S1**: (a) α‐SMA immunostaining (×20) showing strong positivity. (b) S‐100 immunostaining (×20) showing negative staining. (c) CD34 immunostaining (×20) showing negative staining.

## Data Availability

All data supporting the findings of this study are available within the article.
