# Supplementary figures and images for: Gastric Metastasis of Uterine Leiomyosarcoma Detected During Surveillance Endoscopy and Resected by Endoscopic Submucosal Dissection After Multiple Metachronous Metastases: A Case Report
Source: DEN Open. 2026 Jul 7;7(1):e70370. doi: 10.1002/deo2.70370 (PMC13339713; doi:10.1002/deo2.70370)

# Figure S1

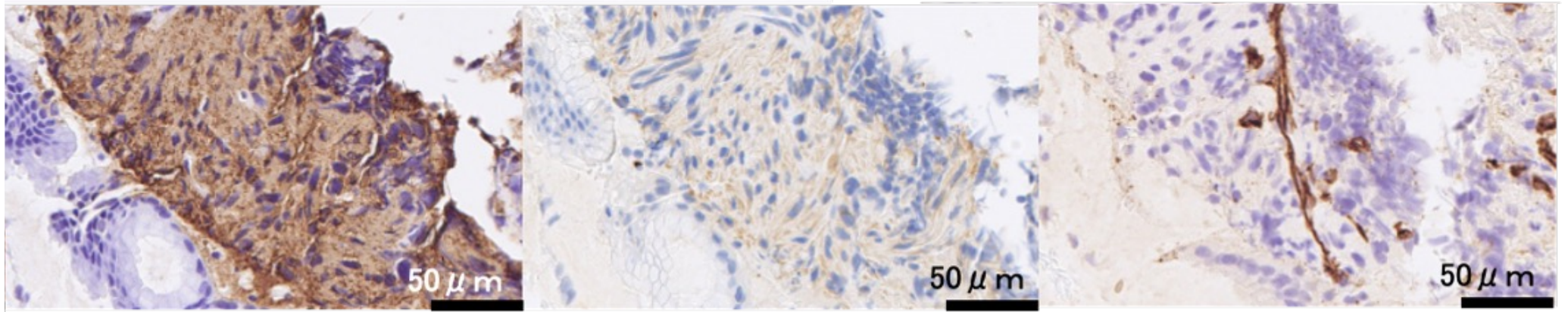

a | b | c

Supplement: Supplementary file 1 — Figure S1: (a) α‐SMA immunostaining (×20) showing strong positivity. (b) S‐100 immunostaining (×20) showing negative staining. (c) CD34 immunostaining (×20) showing negative staining. [file DEO2-7-e70370-s001.pdf]
